# Domain Analysis Reveals That a Deubiquitinating Enzyme USP13 Performs Non-Activating Catalysis for Lys63-Linked Polyubiquitin

**DOI:** 10.1371/journal.pone.0029362

**Published:** 2011-12-28

**Authors:** Yu-Hang Zhang, Chen-Jie Zhou, Zi-Ren Zhou, Ai-Xin Song, Hong-Yu Hu

**Affiliations:** State Key Laboratory of Molecular Biology, Institute of Biochemistry and Cell Biology, Shanghai Institutes for Biological Sciences, Chinese Academy of Sciences, Shanghai, China; Institute of Enzymology of the Hungarian Academy of Science, Hungary

## Abstract

Deubiquitination is a reverse process of cellular ubiquitination important for many biological events. Ubiquitin (Ub)-specific protease 13 (USP13) is an ortholog of USP5 implicated in catalyzing hydrolysis of various Ub chains, but its enzymatic properties and catalytic regulation remain to be explored. Here we report studies of the roles of the Ub-binding domains of USP13 in regulatory catalysis by biochemical and NMR structural approaches. Our data demonstrate that USP13, distinct from USP5, exhibits a weak deubiquitinating activity preferring to Lys63-linked polyubiquitin (K63-polyUb) in a non-activation manner. The zinc finger (ZnF) domain of USP13 shares a similar fold with that of USP5, but it cannot bind with Ub, so that USP13 has lost its ability to be activated by free Ub. Substitution of the ZnF domain with that of USP5 confers USP13 the property of catalytic activation. The tandem Ub-associated (UBA) domains of USP13 can bind with different types of diUb but preferentially with K63-linked, providing a possible explanation for the weak activity preferring to K63-polyUb. USP13 can also regulate the protein level of CD3δ in cells, probably depending on its weak deubiquitinating activity and the Ub-binding properties of the UBA domains. Thus, the non-activating catalysis of USP13 for K63-polyUb chains implies that it may function differently from USP5 in cellular deubiquitination processes.

## Introduction

Ubiquitination is one of the most important post-translational modification processes in eukaryotes [1], [2], [3]. Many different cellular pathways, such as protein degradation, DNA repair, cell cycle progression and apoptosis, are regulated by different specifically linked ubiquitin (Ub) chains [4], [5], [6]. Ub modification is generally accomplished by E1-E2-E3 cascade (sometimes including E4) [2], while these processes can also be reversed by deubiquitinating enzymes (DUB) [7], [8].

There are five families of DUBs in eukaryotic cells including Ub C-terminal hydrolases (UCH), Ub specific proteases (USP), ovarian tumor proteases (OTU), Machado-Joseph disease proteases (MJD) and JAB1/MPN/Mov34 metalloenzymes (JAMM); each group exhibits different function *in vivo* [9], [10], [11]. Besides the essential catalytic core domain, there are a variety of functional domains in DUBs especially in USPs [9]. Bioinformatics analysis indicates that USPs include a catalytic core domain and some other functional domains flanking or inserting in the core domain [12]. Such domains like UBA (Ub associated), ZnF (zinc finger), UIM (Ub interacting motif) play very important functions in USPs [13]. The UBA and UIM domains usually facilitate substrate binding onto the enzymes, while the ZnF domain might be an activator to stimulate the hydrolysis reaction or a regulator to orchestrate other biological functions [14]. However, the catalytic function and the regulation mechanism of these domains in the USP proteases remain to be defined.

USP5, also called isopeptidase T (isoT), is a well-characterized member in USP family [15], [16], [17]. The previous work indicated that USP5 has four Ub-binding sites including two UBA domains, a ZnF domain and a catalytic domain [16]. The tandem UBA domains associate with the distal Ub units when USP5 catalyzes the hydrolysis of polyUb chains [18]. ZnF is the activation domain of USP5 that is recognized by the C-terminal extension of Ub [19], [20]. The catalytic domain consists of the C-box and H-box lobes including substrate binding sites. The function of USP5 is to recognize and recycle the unanchored polyUb chain to keep the free Ub pool stable *in vivo* [21]. As known, the K63-, K48-, K11-, K29- linked and linear polyUb chains are all hydrolyzed by USP5 [22], [23].

USP13 (isoT3) is an ortholog of USP5 in human genome, which shares about 80% sequence similarity with USP5. The *usp13* gene is located in a different chromosome from *usp5* and also represents a different expression profile [24]. It was reported that the mRNA level of USP13 is increased in thyroid tumor pathologies [25], and the activity of USP13 is up-regulated by mitogen activation or virus infection [26]. Also, USP13 can covalently link with an ISG15 suicide substrate, ISG15VS [27]. Although USP13 may have interactions with MURF-1 together with Ubc9, an E2 of SUMO, there is still no valid evidence that indicates USP13 is a SUMO modifying enzyme [28]. From interaction profile analysis, USP13 was implicated in association with some components of P97/VCP complex, a key chaperone in endoplasmic reticulum-associated degradation (ERAD) [29]. Nevertheless, a general hypothesis is that USP13 might share the similar function with USP5 in deubiquitination [9].

To understand the biological function of USP13, we studied different Ub-binding domains (UBDs) in USP13 and compared them with those in USP5 by biochemical and structural approaches. We herewith report the roles of the Ub-binding domains of USP13 in its substrate recognition, enzymatic catalysis and regulation. Different from USP5 in both catalytic activity and activation, USP13 is a novel deubiquitinating enzyme with non-activating catalysis but preferentially for K63-linked polyUb substrates.

## Results

### USP13 prefers to bind with Ub

**Figure 1:**
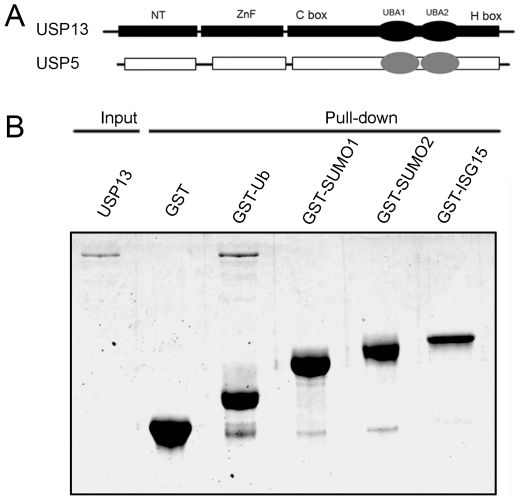
USP13 prefers to bind with Ub. *A*, Domain architectures of USP5 and USP13. NT, N-terminal domain; ZnF, zinc-finger domain; UBA, Ub associated domain; C-box and H-box, two separated lobes of the catalytic domain. *B*, GST pull-down analysis of USP13 (purified with His_6_ tag from *E.coli*) with GST-fused Ub and other UbL proteins. 50% USP13 was loaded as a control. The samples were analyzed by SDS-PAGE with Coomassie blue staining.

USP13 is an ortholog of the well-characterized USP5 enzyme, which presents a relatively high deubiquitinating activity and Ub activation. Sequence analysis shows that these two deubiquitinating enzymes share ∼80% similarity and the same domain architecture including ZnF domain, catalytic C-box and H-box, and a two-UBA insertion (Fig. 1A). The previous study proposed that USP13 might have relations to other Ub-like (UbL) proteins, such as ISG15 [27], SUMO1 and SUMO2 [28]. We performed co-immunoprecipitation (co-IP) experiment to examine whether USP13 recognizes Ub or the UbL proteins. The data show that the C345A mutant (the catalytically inactive form) of USP13 can efficiently precipitate Ub chains, whereas it cannot do for ISG15, SUMO1 and SUMO2 (Fig. S1). The GST pull-down experiment indicates that full-length USP13 can bind with Ub, but not with ISG15, SUMO1 and SUMO2 (Fig. 1B). This suggests that USP13 prefers to bind with Ub chains or ubiquitinated proteins, implicating that it functions as a deubiquitinating enzyme for processing cellular Ub chains or ubiquitinated substrates.

### USP13 possesses a very weak deubiquitinating activity

**Figure 2:**
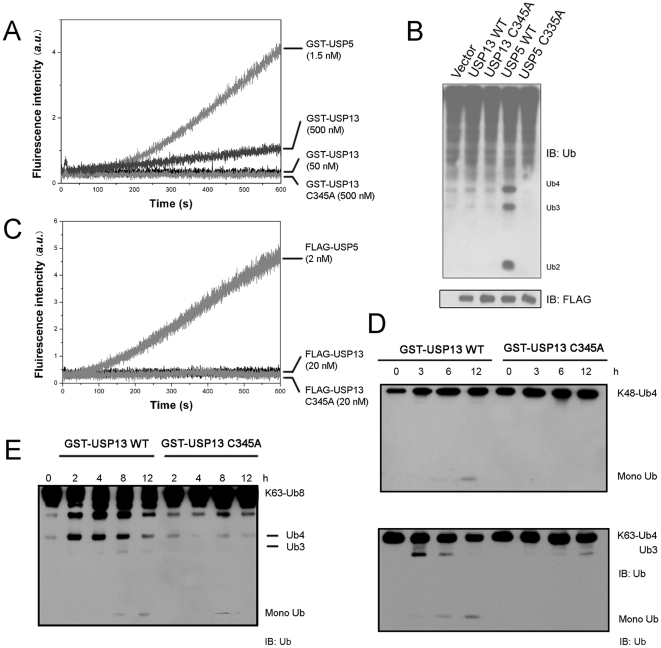
USP13 exhibits weak deubiquitinating activity for K63-linked polyUb chains. *A*, Hydrolysis of Ub-AMC catalyzed by recombinant GST-tagged USP13 or USP5. Ub-AMC (250 nM) was incubated with USP13 (50 or 500 nM) or USP5 (1.5 nM) or USP13-C345A (500 nM). *B*, Cellular ubiquitination levels in the presence of various USPs. HEK 293T cells were transfected with vector, FLAG-tagged USP13, USP5 or their variants. USP13-C345A and USP5-C335A are the active-site point mutants. Before harvesting, the cells were incubated with 10 µM MG132 for 10 hrs. Then, the cell lysates were detected by immunoblotting using an anti-Ub antibody. *C*, Hydrolysis of Ub-AMC catalyzed by FLAG-tagged USP13, USP13-C345A and USP5 purified from HEK 293T cells. Ub-AMC (250 nM) was incubated with USP13 (20 nM), USP13-C345A (20 nM) or USP5 (2 nM). *D*, Hydrolysis of the Ub tetramer by GST-fused USP13 and its C345A mutant. GST-USP13 and its mutant C345A (2 µM) were incubated with K48- or K63-linked Ub4 (0.1 µg/µL), and the reaction product in each time point was detected by immunoblotting using an anti-Ub antibody. *E*, As in (*D*), hydrolysis of the Ub octamer by USP13. GST-USP13 (2 µM) was incubated with K63-linked Ub8 (0.1 µg/µL). GST was set as a negative control.

As known, USP5 is a deubiquitinating enzyme, which binds free Ub chains with its four UBDs [16]. To understand the potential regulatory catalysis of USP13 as USP5, we measured the hydrolytic activities of recombinant GST-fused USP13 and USP5 by using Ub-AMC as a substrate [19], [30]. The hydrolytic rate of USP13 for Ub-AMC is much lower than that of USP5 (Fig. 2A). So, high concentration of GST-USP13 (500 nM) is required for fluorescence detection of the enzymatic activity. It was previously reported that USP5 can hydrolyze unanchored Ub chains from proximal unit one by one, until all the isopeptide bonds have been cleaved completely [18], [19], [31]. All the polyUb chains, K48-, K63-linked and linear, can be recognized and hydrolyzed efficiently by USP5 [31]. We further examined hydrolysis of differently linked diUb substrates by USP13 *in vitro*. USP13 shows no detectable activity for the hydrolysis of three types of diUb, whereas USP5 can hydrolyze them efficiently (Fig. S2). To exclude the possibility that the recombinant USP13 has lost its activity during expression and purification, we overexpressed FLAG-tagged USP13 as well as USP5 in HEK 293T cells and detected ubiquitination levels of the cell lysates. The data show that USP5 can hydrolyze polyUb to oligomeric Ub chains (Ub2∼4), whereas USP13 and the two active-site mutants cannot (Fig. 2B). Furthermore, we purified FLAG-tagged USP13, USP13-C345A and USP5 from HEK 293T cells (Fig. S3), and tested their activities using Ub-AMC as a substrate. Similar results were obtained for the enzymes purified from mamanian cells and overexpression in *E. coli* (Fig. 2C). It implies that USP13 exhibits a very low catalytic activity compared with USP5. Thus, USP13 may exert different deubiquitinating function from USP5, and its hydrolytic activity for Ub chains is not as efficient as that of USP5.

### USP13 can catalyze hydrolysis of the K63-linked polyUb substrates *in vitro*

We further examined the catalytic activity of USP13 on the oligomeric Ub chains by using a GST-fused form. The data show that GST-fused USP13 can catalyze hydrolysis of the K63-linked Ub tetramer (K63-Ub4) with a very low activity, giving a product of Ub3 and finally monoUb (Fig. 2D). However, it takes very little action on the K48-Ub4 substrate, only a trace amount of monoUb can be detected at a long time incubation. Mutation of the active-site (C345A) completely abolishes this function for K63-Ub4, suggesting that USP13 possesses a weak hydrolytic activity preferentially for the K63-linked polyUb substrates. We also tested the hydrolytic activity of USP13 on the K63-Ub8 substrate (Fig. 2E). USP13 can hydrolyze K63-Ub8 to smaller oligomers from Ub8 to Ub4 and finally monoUb. Similar results were also obtained from USP13 without a GST tag (Fig. S4), suggesting that the GST tag has little effect on the activity of USP13. Taken together, different from USP5 that hydrolyzes both K48- and K63-linked Ub chains with high efficiency, USP13 can also catalyze hydrolysis of the polyUb substrates but preferentially K63-linked poly Ub with a very low activity.

### The ZnF domain of USP13 cannot bind with Ub

**Figure 3:**
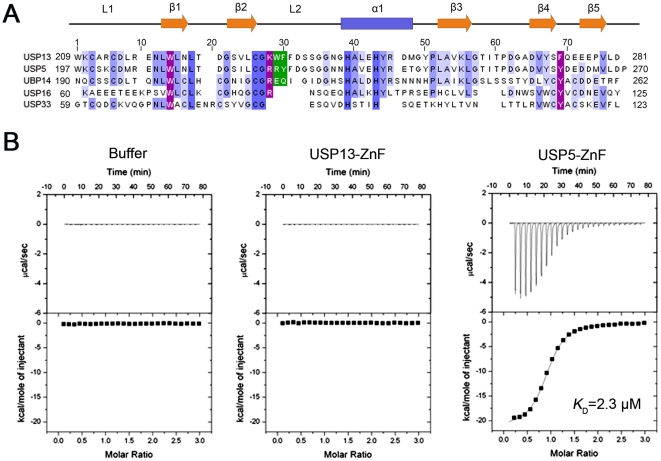
USP13-ZnF cannot bind with Ub but USP5-ZnF can. *A*, Sequence alignment of the ZnF domains from different USPs. The alignment was performed with *ClustalX*. The secondary structures of USP13-ZnF are indicated with α-helices in blue box and β-strands in orange arrows. The most important residues for Ub binding are marked in purple for outside Loop 2 or in green for inside Loop 2. *B*, ITC profiles of USP13-ZnF and USP5-ZnF with Ub. The Ub sample (1 mM stock) was injected into buffer (left), USP13-ZnF (middle) or USP5-ZnF (right) in 25 injections with 8 µL each. The concentrations of USP13-ZnF and USP5-ZnF are 50 µM.

There are four Ub-binding sites in USP5, in which the ZnF domain recognizes the C-terminal diglycine motif of unanchored Ub chains and activates deubiquitination [19]. The sequences of the ZnF domains from these two USPs are homologous (Fig. 3A), suggesting that they share similar structure and Ub-binding property. We firstly examined whether the ZnF domain of USP13 (USP13-ZnF) interacts with Ub by isothermal titration calorimetry (ITC) experiment (Fig. 3B). To our surprise, USP13-ZnF does not bind with Ub, whereas USP5-ZnF binds Ub with a relatively high affinity (*K*_D_ = 2.3 µM). Thus, we suppose that differential Ub recognition of the ZnF domains may provide molecular basis for the different regulatory catalysis of these two enzymes.

### Solution structure of the ZnF domain from USP13

**Figure 4:**
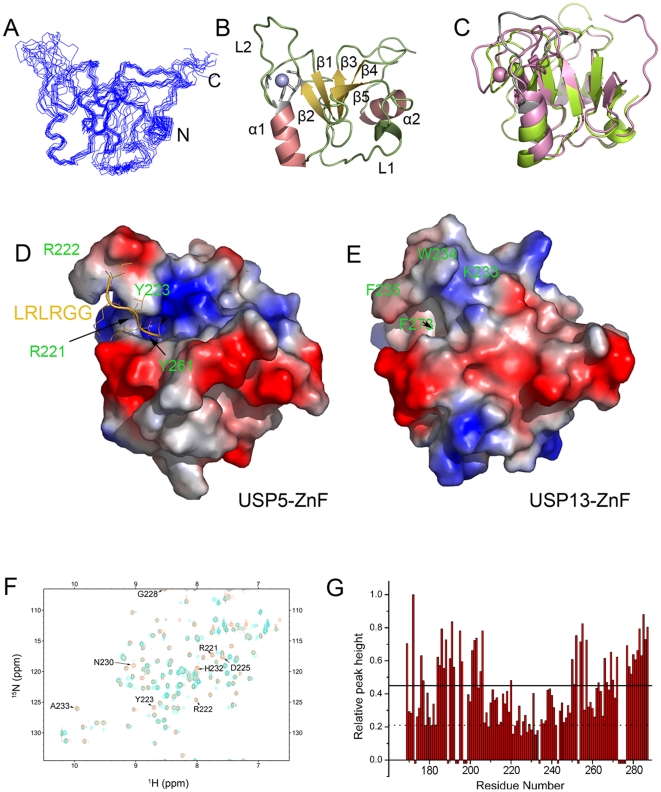
Structure determination of USP13-ZnF and comparison with USP5-ZnF. *A*, Superposition of the backbone traces of 10 representative structures. *B*, Ribbon diagram of a representative structure of USP13-ZnF with secondary structure elements and a zinc ion labeled. The α-helices are colored in red, the β-strands were in yellow, and the loops were in green and the zinc atom is in blue. *C*, Comparison of the structures of USP13-ZnF (lemon) with USP5-ZnF (pink). The L2 loop between β2 and α1, which is not well superposed, is marked in gray. *D*, Electrostatic surface of the USP5-ZnF domain from X-ray crystallography (PDB ID: 2G45). The positive charges are shown in blue and the negative charges are in red. The Ub tail, LRLRGG, is represented by a ribbon (orange) on the structure, and the key residues for the interaction are also labeled on the surface. *E*, Electrostatic surface of the USP13-ZnF domain by NMR analysis. Shown is in the same orientation with in (*D*). The corresponding residues (arrow indicated) in USP5-ZnF and USP13-ZnF responsible for the interactions are also labeled on the surface. All the structures were generated and displayed by PyMol. *F*, Overlay of the ^1^H-^15^N HSQC spectra of USP5-ZnF in the absence (red) and presence (cyan) of Ub (molar ratio, 1: 0.5). *G*, Plot of the relative peak heights of amides against the residue number of USP5-ZnF. The peak intensities were normalized to those without addition of Ub. The bars in negative denote prolines or unassigned residues. The solid and dashed lines indicate the values of *Mean* and *Mean-SD* for the changes of peak heights.

To clarify different catalytic properties based on the two ZnF domains, we elucidated the solution structure of USP13-ZnF by heteronuclear NMR techniques (Fig. 4, Table S1). As predicted from sequence alignment with that of USP5, there is only one zinc ion in the ZnF domain, which was also evidenced by mass spectrometry (Fig. S5). USP13-ZnF contains a zinc core that coordinates with the peptide chain in a C3H pattern (Fig. 4B). The overall solution structure is generally comprised of a β-sheet of five strands flanked by two α-helices (Fig. 4A & 4B), forming an α/β sandwich fold as that of USP5-ZnF (Fig. 4C). Obviously, there is a flexible loop between β2 and α1 (as referred to L2 loop) (Fig. 3A). This domain fold is also similar to those in USP33 [32] and USP16 [33]. Superposition of the backbones reveals no significant difference between the two ZnF structures from USP13 and USP5 (RMSD = 2.1), but the L2-loop regions in the two ZnF domains are a little different (Fig. 4C). Note that the diglycine binding concaves of the two ZnF domains are very distinct, in which the concave of USP5-ZnF is deeper than that of USP13-ZnF, and its binding surface contains more positive charges (Fig. 4D & 4E). Thus, comparison of the ZnF-domain structures provides implication for the distinct function of these two USP enzymes.

### Mechanistic insights into the Ub binding of the ZnF domains

In the ZnF domain of USP5, three residues, W209, R221 and Y261, play critical roles in catalytic activation [19]. Sequence alignment of the ZnF domains indicates that some residues in USP5-ZnF, which are different in USP13-ZnF, might be involved in Ub binding (Fig. 3A). From chemical shift perturbation of USP5-ZnF upon binding with Ub, the residues around ^221^RRY motif in L2 loop are largely perturbed (Fig. 4F & 4G). We selected four critical residues in USP5-ZnF that are largely perturbed when titrated with Ub and variable in USP13-ZnF, and substituted them with the corresponding ones of USP13-ZnF. The ITC experiment indicates that the single mutations of USP5-ZnF, especially R221K and Y261F, significantly weaken the binding of USP5-ZnF with Ub, while replacement of the ^221^RRY motif with the corresponding residues (^233^KWF) in USP13-ZnF completely abolishes the binding of USP5-ZnF with Ub (Table 1, Fig. S6). Thus, from mutation analysis of the critical residues in USP5-ZnF, we have obtained some valuable information on the novel ZnF domain of USP13 that cannot act as a Ub receptor.

**Table 1:** Dissociation constants of wild-type USP5-ZnF and its mutants binding with free Ub.

ZnF domain	*K*_D_ (M)	stoichiometry
WT	(2.3±0.0)×10^−6^	0.90
R221K	(1.0±0.1)×10^−4^	1.54
Y261F	(8.8±0.7)×10^−5^	1.22
Y223F	(6.4±0.2)×10^−6^	1.26
R222W	(9.1±0.2)×10^−6^	1.02
R222W/Y223F	(1.6±0.0)×10^−5^	0.94
R221K/Y223F/R222W	N.D.	N.D.

N.D., not detectable.

### Domain swapping confers USP13 the ability to be activated by Ub

**Figure 5:**
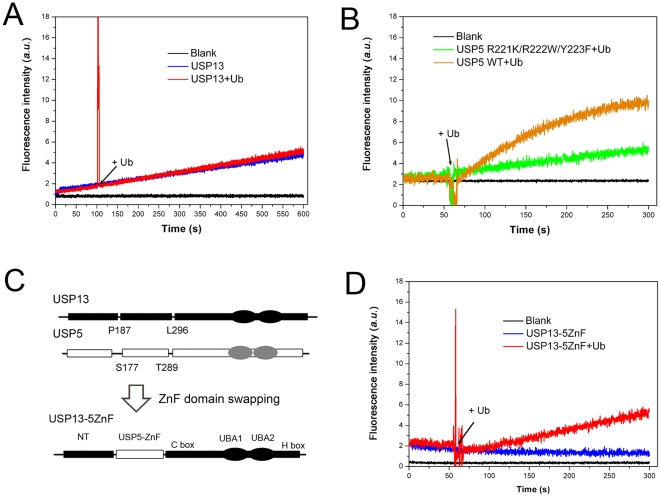
Gain of the catalytic activation of USP13 through ZnF-domain swapping from USP5. *A*, Non-activating catalysis of USP13 by free Ub. Ub-AMC (250 nM) was incubated with GST-USP13 (1 µM) for 100 s, and then Ub (38 µM) was added into the reaction mixture. *B*, Activating catalysis of USP5 by free Ub. In comparison with wild-type USP5 that exhibits high Ub activation, the USP5 mutant (R221K/R222W/Y223F) loses the property of being activated by Ub. Ub-AMC (250 nM) was incubated with USP5 or its mutant (10 nM) for 50 s, and then Ub (100 nM) was added into the reaction mixture. *C*, Schematic diagram of the domain-swapped mutant of USP13. USP13-5ZnF denotes a chimera enzyme of USP13 with a substituted ZnF domain from USP5. *D*, USP13-5ZnF can be activated by free Ub. Ub-AMC (250 nM) was incubated with USP13-5ZnF (100 nM) for 50 s, and then Ub (1 µM) was added into the reaction mixture. All the reactions were monitored by fluorescence of released AMC at 460 nm.

Since the ZnF domain of USP13 is not the Ub receptor, it is crucial to examine whether USP13 could be catalytically activated by Ub. We performed deubiquitination assays using Ub-AMC as a substrate [19], [30]. In the presence of Ub, there is no catalytic activation observed in USP13 (Fig. 5A). On the contrary, USP5 exhibits strong Ub activation (Fig. 5B). The ZnF triple mutant of USP5 (R221K/R222W/Y223F), which cannot bind with Ub (Fig. S6), exhibits loss of the activation. To confirm the non-activating catalysis of USP13 through its ZnF domain, we generated a chimera enzyme by ZnF-domain swapping (Fig. 5C). As expected, the USP5-ZnF substituted USP13 (USP13-5ZnF) presents only a detectable activity, but it returns to be activated upon addition of Ub (Fig. 5D). It suggests that the ZnF domain in USP5 is a Ub receptor responsible for activation of deubiquitination, whereas that in USP13 is not. Taken together, besides the catalytic domains that define the hydrolysis efficiency, USP13 can catalyze hydrolysis of the K63-linked polyUb substrates but with a very low activity.

### The tandem UBA domains of USP13 interact with Ub

**Figure 6:**
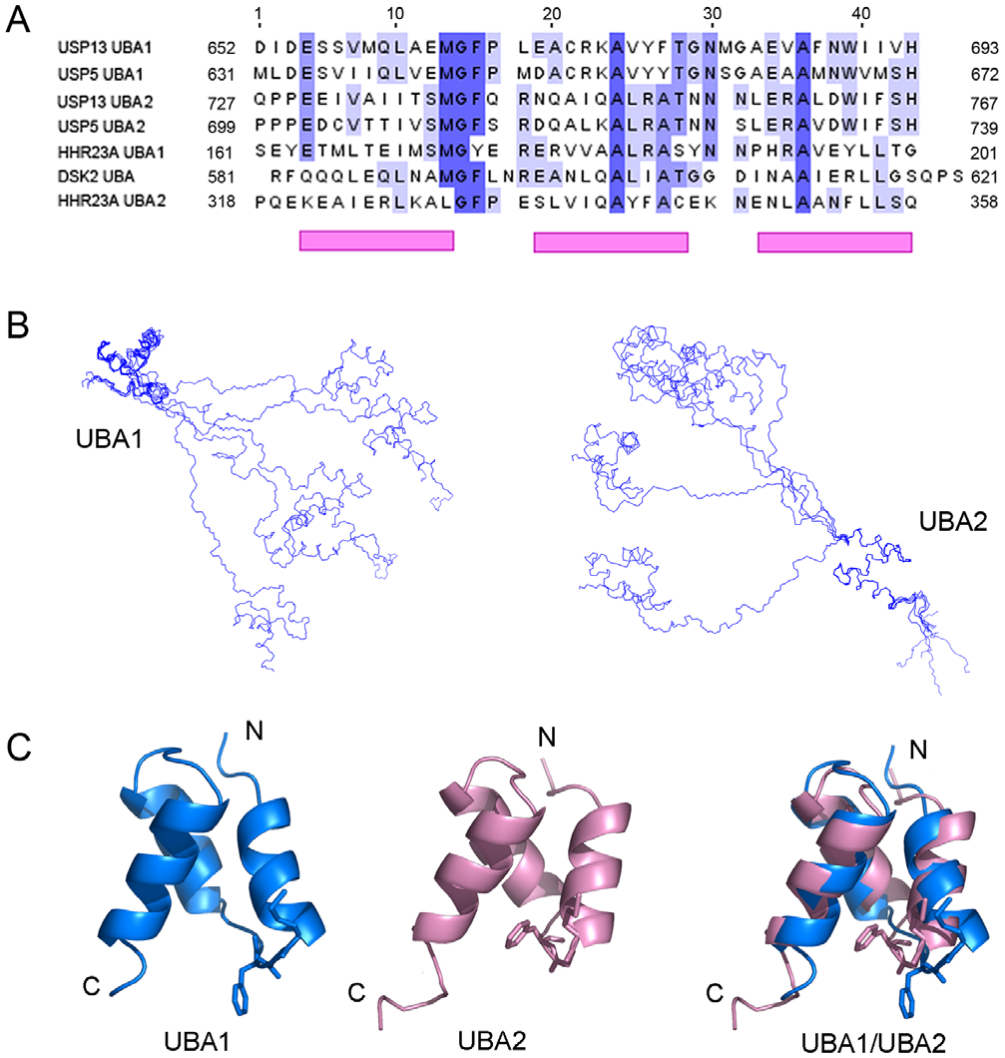
Solution structure of the tandem UBA12 domain of USP13. *A*, Sequence alignment of the two UBA domains of USP13 with some classical UBA domains. The alignment was performed with *ClustalX*. The pink boxes denote α-helices, and the MGF motif is conserved in all UBAs. *B*, Ensemble of 5 structures superimposed on the polypeptide backbones of UBA1 or UBA2. The graphs were generated by using MOLMOL. *C*, Ribbon representation of the solution structures of UBA1 and UBA2, and their comparison. The structures were generated and displayed by PyMol.

Both USP13 and USP5 contain tandem UBA domains inserted between the C-box and H-box lobes (Fig. 1A), and the two UBA domains are conserved in sequence with a canonical MGF motif (Fig. 6A) [23], [34]. We firstly solved the solution structure of the tandem UBA domains of USP13 (USP13-UBA12) (Table S1). There is no NOE correlation between the UBA domains, indicating that these two domains have no direct contact due to a long flexible linker (Fig. 6B). As usual, both domains hold a canonical three-helix bundle fold with a MGF motif (Fig. 6C), suggesting that the tandem UBA domains from USP13 and USP5 may have similar Ub-binding properties.

**Figure 7:**
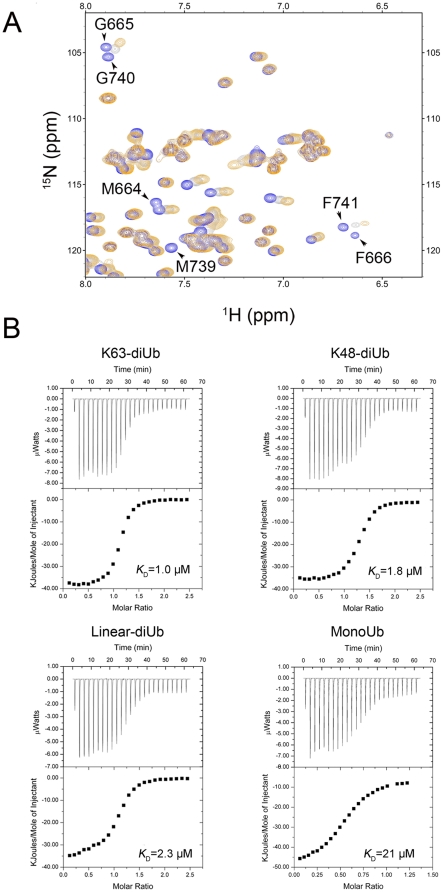
Binding of USP13-UBA12 with various types of diUb chains. *A*, NMR titration for mapping the binding surface on USP13-UBA12. Shown is the overlay of ^1^H-^15^N HSQC spectra of USP13-UBA12 in the absence (blue) or presence of increasing amounts of Ub (gray, 1: 0.6; orange, 1: 1). The residues on the classical binding patch are labeled in the spectra. *B*, ITC profiles of USP13-UBA12 with various types of diUb or monoUb. The UBA12 samples (1.25 mM stock) were injected into K63-, K48-, linear diUb (0.1 mM) or monoUb (0.2 mM) in 25 injections with 1.5 µL each.

We then prepared tandem and individual UBA domains and performed GST pull-down experiments. As a result, USP13-UBA12 can specifically interact with Ub but not with other UbL proteins (Fig. S7A). Moreover, the individual UBA domains (UBA1 and UBA2) of USP13 can also bind with Ub (Fig. S7B). NMR titration further demonstrates that USP13-UBA12 interacts with Ub in a canonical manner (Fig. 7A). As a control, USP13-UBA12 cannot interact with ISG15 (Fig. S8), which was previously proposed to be potential [27]. The chemical shifts of M664, F666, M739 and F741 on USP13-UBA12 are largely perturbed when titrated with Ub. These residues mainly comprise the classical site for Ub binding [23], [34], [35], [36]. This means that the interaction of USP13-UBA12 with Ub adopts an similar pattern with that of USP5-UBA12 [18], suggesting that the tandem UBA domains are the Ub receptors for their catalytic function in the two USP enzymes.

We also performed ITC experiment on the interactions of USP13-UBA12 with different types of diUb. As predicted, USP13-UBA12 binds various types of diUb some ten-fold stronger than monoUb. The binding affinities for USP13-UBA12 with diUb are around a micromolar scale, while it is preferential for K63-diUb (*K*_D_ = 1 µM). So, we assume that it is a reasonable explanation for USP13 that performs a weak catalysis preferentially for the K63-linked polyUb substrates.

### USP13 regulates cellular levels of CD3δ through its UBA domains and weak hydrolytic activity

**Figure 8:**
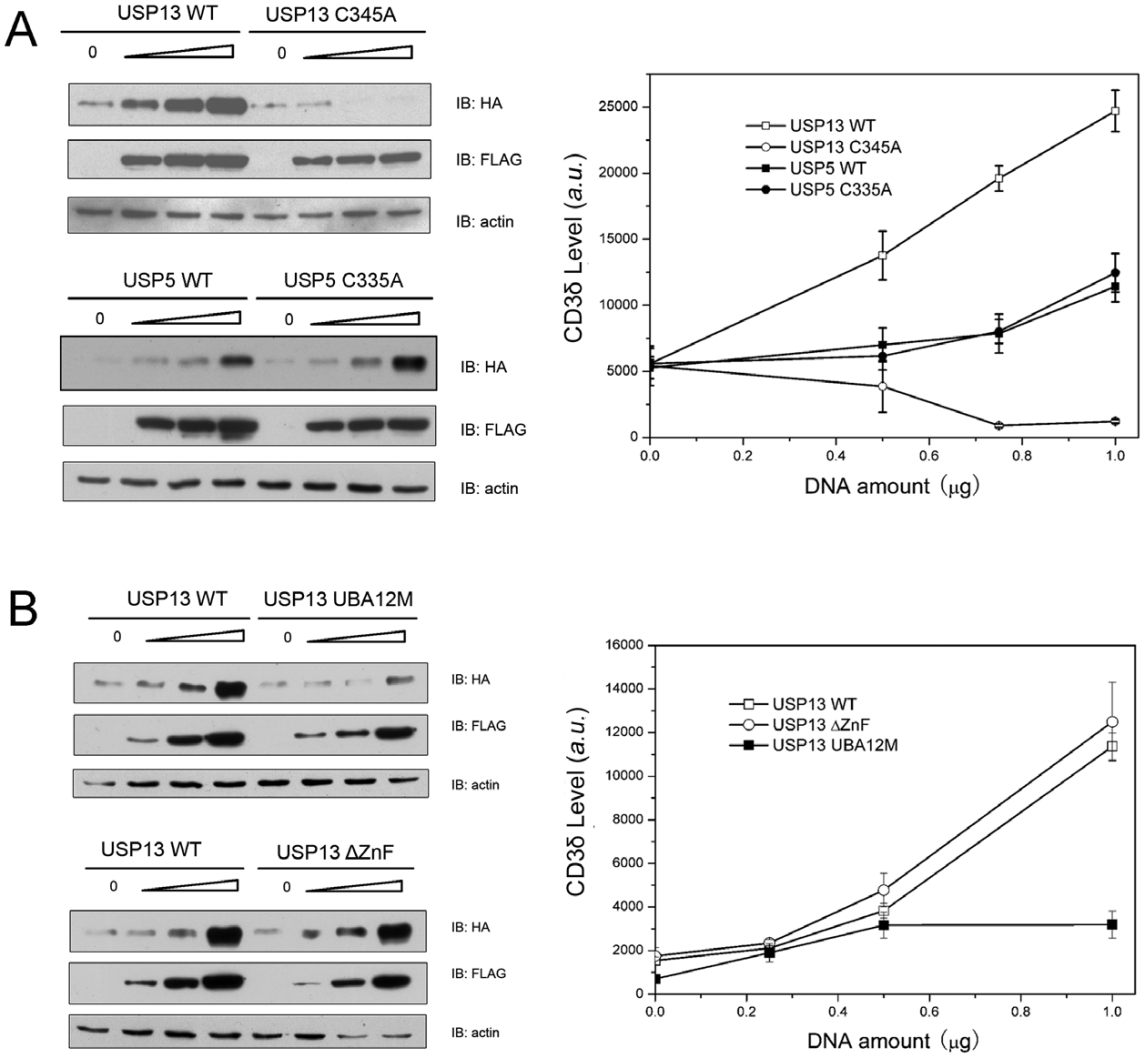
USP13 regulates cellular levels of CD3δ. *A*, Dose-dependent experiments for USP13 or USP5 and their mutants on the CD3δ levels. HEK 293T cells were co-transfected with equal amount (1 µg) of CD3δ-HA and increasing amount (0.5, 0.75 and 1.0 µg) of FLAG-tagged USP13, USP13-C345A, USP5 or USP5-C345A. Cells were harvested and lysed 36 hrs after transfection, and then the samples were analyzed by Western blotting. Data analysis (n = 3) for the amount of CD3δ versus amount of the transfected DNA of USP13, USP5 or their mutants using western blotting and quantified by the intensity of western blotting. *B*, Effects of the UBA12 mutant or ZnF-deletion mutant of USP13 on the CD3δ levels. USP13-UBA12M is the mutant with two point mutations in each UBA domain (M664E/M739E). USP13-ΔZnF is the mutant with deletion of the ZnF domain. Data analysis was performed as indicated in *A*.

Since USP13 possesses the weak activity for K63-polyUb but without Ub activation and this enzyme might associate with some components of P97/VCP complex [29]. We examined the potential role of USP13 in regulating cellular protein levels by using CD3δ, a classical ERAD substrate as a model [37], [38], [39]. As a result, USP13 can significantly increase the protein level of CD3δ, whereas its C345A mutant loses its ability (Fig. 8A). On the contrary, the C335A mutation of USP5 has similar increasing effect on the CD3δ level with the wild-type USP5. Mutation in the UBA domains of USP13, which blocks Ub binding [18], [34], significantly attenuates this increasing effect (Fig. 8B). However, deletion of the ZnF domain in USP13 has little effect on the CD3δ level (Fig. 8B). This suggests that both the active site and the tandem UBA domains are involved in this process, providing an implication that the deubiquitinating activity of USP13 and its Ub-binding ability of the UBA domains play important roles in regulating the cellular protein levels.

## Discussion

USP13 is an ortholog of the well-characterized USP5 enzyme implicated in deubiquitination of cellular ubiquitinated substrates. However, our studies indicate that USP13 is very different from USP5 both in catalytic efficiency and substrate preference. The activity of USP5 is dramatically stimulated by Ub, a product of deubiquitination [19]. USP13 retains only a basal catalytic activity for K63-polyUb, and it cannot be activated by the reaction product. This study by analyzing the UBDs provides compelling evidence for the regulatory catalysis of deubiquitination of USP13 and potentially for mediating protein levels in cells.

### The ZnF domain of USP13 is defective in Ub binding and catalytic activation

There are many ZnF domains in USPs in human genome, which share similar sequence and adopt the same fold [14], [40]. Up to date, three ZnF-UBP domains from USP5, HDAC6 and USP16 have been proved to bind with Ub in the C-terminal LRLRGG region [18]. Sequence alignment shows that the most important residues for Ub binding are really conserved in all of the ZnF domains (Fig. 3A). However, a few residues in USP13-ZnF are varied especially in the L2-loop region, in which R221 and Y261 in USP5-ZnF are the key residues for Ub binding and catalytic activation [19]. From the solution structure of USP13-ZnF, there are two reasons that make it unable to bind with Ub. One is the relatively shallow concave for accepting the C-terminal extension of Ub and the less positive charges around the concave. The other is no hydrogen bond formed by W234 with other residues or F235 with R74 of Ub, in contrast to USP5-ZnF where some hydrogen bonds are formed by the corresponding R222 with G229 and Y223 with R74 of Ub. This partly explains the importance of the residues flanking the key residues on the binding surface of the L2 loop. Deletion of the ZnF domain in USP13 has little impact on regulation of the CD3δ level. Although there are lots of ZnF domains adopting the similar fold in DUBs or other proteins, it does not mean that all the ZnF domains reserve the ability to bind with Ub. The ZnF domain of USP33 is much similar in both sequence and structure with that of USP16, but it cannot bind with Ub [19], [33], [41]. Thus, the defective ZnF domain of USP13 may have yet unidentified biological function in eukaryotic cells, but this still remains to be explored.

### Function of the UBA domains in USP13

UBA is a common domain found in many proteins [13], [42]. Four major groups of UBA are classified according to their polyUb interaction preference [23]. Although the previous study proposed that the tandem UBA domains of USP13 have a potential to bind with ISG15 [27], our data from GST pull-down and NMR titration exclude this possibility (Fig. S7A & S8). Even by using NMR titration, a more sensitive technique for detecting weak interaction, there is no sign of interaction between ISG15 and USP13-UBA12. It is reported that the tandem UBA domains of USP13 are Ub specific receptors, because both domains share a canonical MGF motif for Ub binding [23], [34]. Moreover, interaction of the individual UBA1 domain from USP5 with Ub tetramer is not as efficient as UBA2 [23]; but in the context of the full-length enzyme, interaction of the UBA1 moiety can also be detected [18]. For USP13, both UBA1 and UBA2 can bind with Ub (Fig. S6B); while the single UBA1 of USP13 was not stable enough to be well expressed and purified. Interestingly, the binding of USP13-UBA12 with K63-diUb is a bit stronger than K48- or linear diUb, although linear diUb seems to adopt a similar open fold with the K63-linked but different from the K48-linked. Thus, we conclude that the tandem UBA domains of both USP5 and USP13 prefer to bind with the polyUb chains, so as to increase the hydrolysis efficiency.

### Biological implication of the weak catalytic activity of USP13 for polyUb chains

As ubiquitination, deubiquitination is also implicated in controlling protein degradation in cells [9], [10], [37]. Although USP13 is an ortholog of USP5, it shows only a very low basal deubiquitination activity and loss of Ub activation, due to the inefficient catalytic domain and the defective ZnF domain. By co-transfection of USP13 and CD3δ, the cellular level of CD3δ is remarkably regulated by USP13, and the tandem UBA domains may be involved in this process (Fig. 8). We propose that USP13 can regulate deubiquitination of the ubiquitinated proteins and thus retards their degradation in cells. So, elucidating how USP13 recognizes ubiquitinated proteins is the prerequisite for understanding the mechanism of cellular proteostasis orchestration.

Since the overall structure of USP13 is not elucidated yet, the three-dimensional structure of USP5 in complex with Ub (PDB ID: 3IHP) reveals that the ZnF domain is located beside the catalytic core. It means that the proximal Ub moieties would be fixed together by the active ZnF domain. This is evidenced by ZnF domain-swapped USP13 that the activation is recovered (Fig. 5D). One possible explanation is that the entire enzyme undergoes conformational changes especially at the catalytic triplet, when it binds with Ub chains. For many DUBs, such as USP14 [43], USP1 [44] and USP4 [45], due to no proper activation, the catalytic activity *in vitro* is relatively low [9]. It seems that the ZnF domain of USP5 refines the enzyme conformation to achieve catalytic activation. However, USP13 may have lost this effect during the evolution, because there is no such an ortholog in yeast.

USP5 is generally recognized as a deubiquitinating enzyme for trimming unanchored polyUb chains, and it may play a role in maintaining the homeostasis of the free Ub pool [10]. Our study demonstrates that USP13 cannot hydrolyze polyUb chains to free Ub molecules efficiently, and it is unlikely to act as a regulator for the free Ub pool. Recent studies suggested that USP13, as a deubiquitinase, can stabilize and regulate MITF and Siah2 levels in cells [46], [47], while the deubiquitination process of USP13 can also be orchestrated by beclin-1 [48]. Altogether, different from USP5, USP13 may play important roles in regulating deubiquitination and stability of some cellular protein substrates.

## Materials and Methods

### Constructs and protein purification

The genes encoding USP13 and USP5 were cloned from the cDNA library of HEK 293T cell. The plasmids for expressing the GST-fused proteins, Ub, ISG15, SUMO1 and SUMO2, USP13 (residues 21–863), USP5, and their active-site mutants (USP13-C345A, USP5-C335A) were generated by inserting the cDNA into a pGEX-4T-3 vector. The genes for USP13 (residues 21–863), USP13-UBA12 (652–776) were cloned into pET-32M, and those for USP13-ZnF (178–301), USP5, USP5-ZnF (169–289) and their point mutants were cloned into pET-M. The expression vector for chimera protein USP13-5ZnF was constructed by PCR and inserted into pET-32M plasmid. All proteins were expressed in *E. coli* BL21 and purified with Ni-NTA agarose or glutathione agarose. The His6 and Trx tags were removed by thrombin cleavage on the column and the proteins were further purified by size-exclusion chromatography (Fig. S9). The concentrations of all the proteins were determined spectrophotometrically at 280 nm with the extinction coefficient of each protein. The genes for USP13, USP5, and their point mutants (C345A, C335A, M664E/M739E) and the ZnF deletion (USP13-ΔZnF, deletion of 190–292) were also cloned into pCMV-Tag2B for expression in mammalian cells. FLAG tagged USP13, USP13-C345A and USP5 are purified by immunoprecipitation and eluted by 1× FLAG peptide (0.1 mg/mL, SIGMA), then quantified against GST-USP13 by coomassie blue staining.

### Cell culture, transfection and Western blotting

HEK 293T cells (American Type Culture Collection, Manassas, VA, USA) were cultured in DMEM containing 10% FBS. All the cells were transfected with the indicated constructs using FuGENE HD Transfection Reagent (Roche). About 36 hrs after transfection, the cells were harvested and lysed in the Triton X-100 lysis buffer. The modified proteins in the cell lysates were detected by Western blotting using anti-Ub, anti-ISG15, anti-SUMO1 and anti-SUMO2 antibodies (Santa Cruz).

### GST pull-down experiment

GST-fused Ub, ISG15, SUMO1 or SUMO2 was loaded on glutathione agarose, then USP13-UBA12 (∼100 µM) was added. After 1-hr incubation, the beads were washed for three times and eluted by GSH buffer, and then the samples were subjected to SDS-PAGE with Coomassie blue staining.

### Structure determination

The ^15^N/^13^C-labeled USP13-ZnF or USP13-UBA12 sample (∼0.8 mM) was dissolved in a buffer containing 20 mM phosphate, 50 mM NaCl and 0.01% NaN3, pH6.5. All of the heteronuclear spectra were recorded at 25°C on a Bruker Avance 600-MHz spectrometer. The backbone and side-chain assignments were completed by analyzing the following spectra as previously described [49]. Total dihedral angle restraints (Φ/Ψ) were obtained from chemical shifts of ^1^Hα, ^13^Cα, ^13^Cβ, and ^13^CO using TALOS. Three-dimensional ^15^N- and ^13^C-edited nuclear Overhauser effect spectra were acquired to provide the distance restraints for structure computation by using ARIA2.0. A family of 200 structures was calculated using the simulated annealing protocol, and 15 of the lowest energy structures were selected. Structure assessment was performed by PROCHECK. All structures were generated using MolMol or PyMol as indicated in the figures.

### NMR titration and isothermal titration calorimetry (ITC)

All NMR titration experiments were carried out in a buffer containing 20 mM phosphate, 50 mM NaCl, pH6.5. ^15^N-labeled USP13-UBA12, USP13-ZnF and USP5-ZnF (∼0.2 mM) were titrated with Ub, and then the ^1^H-^15^N HSQC spectra were recorded. The ZnF or UBA12 samples for ITC experiments were dialyzed in a Tris-HCl buffer (50 mM Tris, 150 mM NaCl, pH 7.5). DTT (1 mM) was also included in the buffer for titration of ZnF. The ITC experiments were performed on a Microcal VP-ITC or Microcal iTC_200_ at 25°C.

### Deubiquitination assays

All the experiments for hydrolysis of Ub chains or Ub-AMC were performed in a buffer of 50 mM Tris-HCl (pH 8.0), 150 mM NaCl, and 5 mM DTT. K48- and K63-linked diUb were synthesized as reported [50]. The hydrolysis of diUb chains were assayed as follows. K48-, K63- or linear linked diUb (10 µM) was incubated with 1 nM of USP13 or USP5 at 37°C. Each 10-µL sample was taken out at the indicated time point and detected by SDS-PAGE with silver staining. K48-, K63-Ub4 (0.1 µg/µL) and K63-Ub8 (0.1 µg/µL) were purchased from Boston Biochem. The reaction was performed as the diUb experiments. Each 10-uL sample was taken out at indicated time point and detected by Western blotting with an anti-Ub antibody. Ub-AMC (250 nM) was incubated with GST-USP13, GST-USP5, FLAG-USP13, FLAG-USP5 or their mutants at different concentrations. The fluorescence of AMC was recorded on a Fluorescence Spectrophotometer (Varian Cary Eclipse) during the reaction process with an excitation at 380 nm, and the intensities at 460 nm were monitored. Hydrolysis activation of USP5 and USP13 were measured by adding Ub at the indicated time.

### Accession codes

Coordinates and structure factors have been deposited in the Protein Data Bank (PDB) with 2L80 for USP13-ZnF and 2LBC for USP13-UBA12.

## Supporting Information

Figure S1 Co-IP experiments for USP13 binding with Ub or other UbLs. USP13 is able to accumulate Ub chains or ubiquitinated substrates but not with those of other UbLs. IP: anti-FLAG antibody; IB: anti-Ub, anti-ISG15, anti-SUMO1, and anti-SUMO2 antibodies. 50% lysates were loaded as input.
(DOC)

Figure S2 Time courses of the hydrolysis reactions for diUb chains by USP13 or USP5. K48-, K63-linked or linear diUb (10 µM) was incubated with USP13 or USP5 (1 nM). Equivalent sample was taken out at indicated time point and analyzed by SDS-PAGE with silver staining.
(DOC)

Figure S3 Purification of FLAG-tagged enzymes by Immunoprecipitation. FLAG-tagged USP13, USP13-C345A, and USP5 were purified from HEK 293T cells by anti-FLAG beads and eluted with 1× FLAG, and analyzed by SDS-PAGE with Coomassie blue staining.
(DOC)

Figure S4 Time courses of the hydrolysis reactions of USP13 for K48- or K63-linked Ub4 chains. Purified recombinant USP13 (5 µM) were incubated with K48- or K63-linked Ub4 substrate (0.025 µg/µL), and the reaction product in each time point was detected by immunoblotting using an anti-Ub antibody.
(DOC)

Figure S5 Mass spectrometric analysis of the metal ions in USP13-ZnF. *A*, Spectrum of USP13-ZnF in native state. The main peak is at a molecular weight of 12889. *B*, Spectrum of USP13-ZnF in denatured state. The main peak is at a molecular weight of 12823. The peak shift of 66 units (12889–12823) indicates that only one zinc ion (atomic weight of 65) exists in the ZnF domain.
(DOC)

Figure S6 ITC experiments for measuring the binding affinities of different USP5-ZnF mutants with Ub. The mutants include R221K, Y261F, R222W, Y223F, R222W/Y223F and R221K/R222W/Y223F. All the titrations were performed under the same condition. The dissociation constants of the mutants binding with Ub were estimated from the titration curves by a 1∶1 stoichiometry.
(DOC)

Figure S7 GST pull-down experiments for interactions of the UBA domains from USP13 with Ub and other UbLs. *A*, GST pull-down analysis of USP13-UBA12 with GST-fused Ub and other UbL proteins. 50% USP13-UBA12 was loaded as a control. The samples were analyzed by SDS-PAGE with Coomassie blue staining. UBA12 from USP13 bind with Ub but not with other UbLs. *B*, GST pull-down experiment for interactions of the single UBA domains from USP13 with Ub. The GST-fused UBA1 and UBA2 of USP13 were purified from *E. coli* and incubated with Ub for pull-down analysis. The samples were then analyzed by SDS-PAGE with Coomassie blue staining. 50% Ub was loaded as an input.
(DOC)

Figure S8 NMR titration showing that USP13-UBA12 cannot interact with ISG15. Shown is the overlay of ^1^H-^15^N HSQC spectra of USP13-UBA12 (0.2 µM) in the absence (red) or presence (cyan, 1: 8) of ISG15.
(DOC)

Figure S9 Purification of USP13, USP5 and their mutants. GST-fused USP13 and its C345A mutant were purified by glutathione agarose. USP13, USP5, USP13-5ZnF and the ZnF triple mutant of USP5 (R221K/R222W/Y223F) were purified by Ni-NTA agarose, and the Trx-His6 tags were removed by thrombin cleavage at 22°C overnight, and further purified by Superdex-200 (GE Healthcare). Samples were analyzed by SDS-PAGE with Coomassie blue staining. All the proteins are stored at −70°C in a buffer of 50 mM Tris-HCl, 150 mM NaCl and 5 mM DTT (pH 8.0).
(DOC)

Table S1 Experimental restraints and structural statistics of the ZnF and UBA12 domains from USP13.
(DOC)
