# Supplementary material for: Domain Analysis Reveals That a Deubiquitinating Enzyme USP13 Performs Non-Activating Catalysis for Lys63-Linked Polyubiquitin
Source: PLoS One. 2011 Dec 28;6(12):e29362. doi: 10.1371/journal.pone.0029362 (PMC3247260; doi:10.1371/journal.pone.0029362)
Supplement: Figure S1 — Co-IP experiments for USP13 binding with Ub or other UbLs. USP13 is able to accumulate Ub chains or ubiquitinated substrates but not with those of other UbLs. IP: anti-FLAG antibody; IB: anti-Ub, anti-ISG15, anti-SUMO1, and anti-SUMO2 antibodies. 50% lysates were loaded as input. (DOC) [file pone.0029362.s001.doc]

**Figure S1**


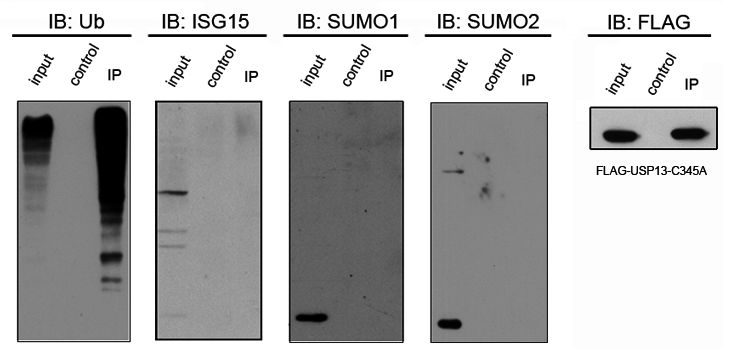


**Figure S1** Co-IP experiments for USP13 binding with Ub or other UbLs. USP13 is able to accumulate Ub chains or ubiquitinated substrates but not with those of other UbLs. IP: anti-FLAG antibody; IB: anti-Ub, anti-ISG15, anti-SUMO1, and anti-SUMO2 antibodies. 50% lysates were loaded as input.
