# Supplementary material for: Domain Analysis Reveals That a Deubiquitinating Enzyme USP13 Performs Non-Activating Catalysis for Lys63-Linked Polyubiquitin
Source: PLoS One. 2011 Dec 28;6(12):e29362. doi: 10.1371/journal.pone.0029362 (PMC3247260; doi:10.1371/journal.pone.0029362)
Supplement: Figure S2 — Time courses of the hydrolysis reactions for diUb chains by USP13 or USP5. K48-, K63-linked or linear diUb (10 µM) was incubated with USP13 or USP5 (1 nM). Equivalent sample was taken out at indicated time point and analyzed by SDS-PAGE with silver staining. (DOC) [file pone.0029362.s002.doc]

**Figure S2**


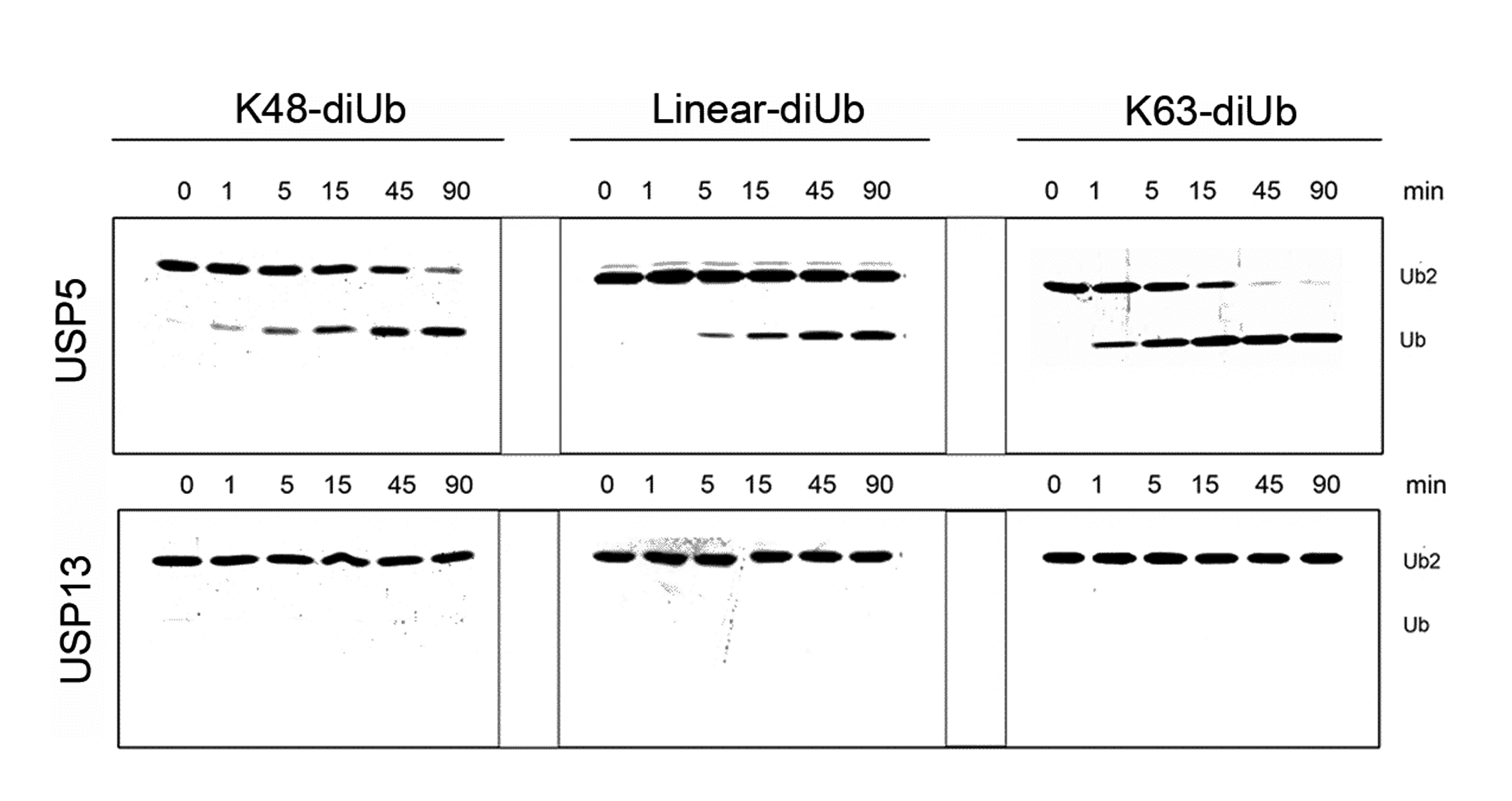


**Figure S2** Time courses of the hydrolysis reactions for diUb chains by USP13 or USP5. K48-, K63-linked or linear diUb (10 μM) was incubated with USP13 or USP5 (1 nM). Equivalent sample was taken out at indicated time point and analyzed by SDS-PAGE with silver staining.
