# Supplementary material for: Domain Analysis Reveals That a Deubiquitinating Enzyme USP13 Performs Non-Activating Catalysis for Lys63-Linked Polyubiquitin
Source: PLoS One. 2011 Dec 28;6(12):e29362. doi: 10.1371/journal.pone.0029362 (PMC3247260; doi:10.1371/journal.pone.0029362)
Supplement: Figure S3 — Purification of FLAG-tagged enzymes by Immunoprecipitation. FLAG-tagged USP13, USP13-C345A, and USP5 were purified from HEK 293T cells by anti-FLAG beads and eluted with 1× FLAG, and analyzed by SDS-PAGE with Coomassie blue staining. (DOC) [file pone.0029362.s003.doc]

**Figure S3**


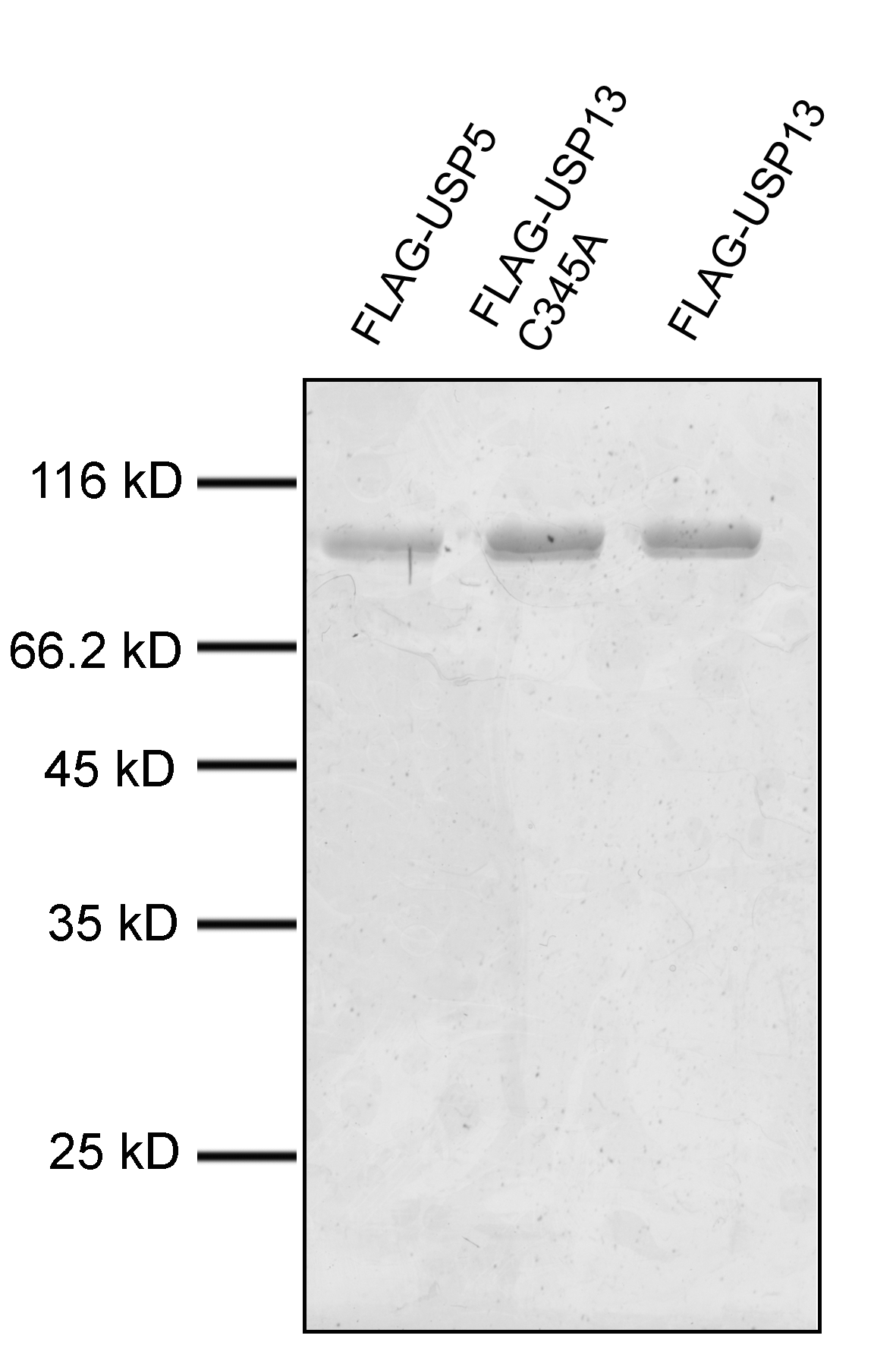


**Figure S3** Purification of FLAG-tagged enzymes by Immunoprecipitation. FLAG-tagged USP13, USP13-C345A, and USP5 were purified from HEK 293T cells by anti-FLAG beads and eluted with 1 x FLAG, and analyzed by SDS-PAGE with Coomassie blue staining.
