# Supplementary material for: Domain Analysis Reveals That a Deubiquitinating Enzyme USP13 Performs Non-Activating Catalysis for Lys63-Linked Polyubiquitin
Source: PLoS One. 2011 Dec 28;6(12):e29362. doi: 10.1371/journal.pone.0029362 (PMC3247260; doi:10.1371/journal.pone.0029362)
Supplement: Figure S4 — Time courses of the hydrolysis reactions of USP13 for K48- or K63-linked Ub4 chains. Purified recombinant USP13 (5 µM) were incubated with K48- or K63-linked Ub4 substrate (0.025 µg/µL), and the reaction product in each time point was detected by immunoblotting using an anti-Ub antibody. (DOC) [file pone.0029362.s004.doc]

**Figure S4**


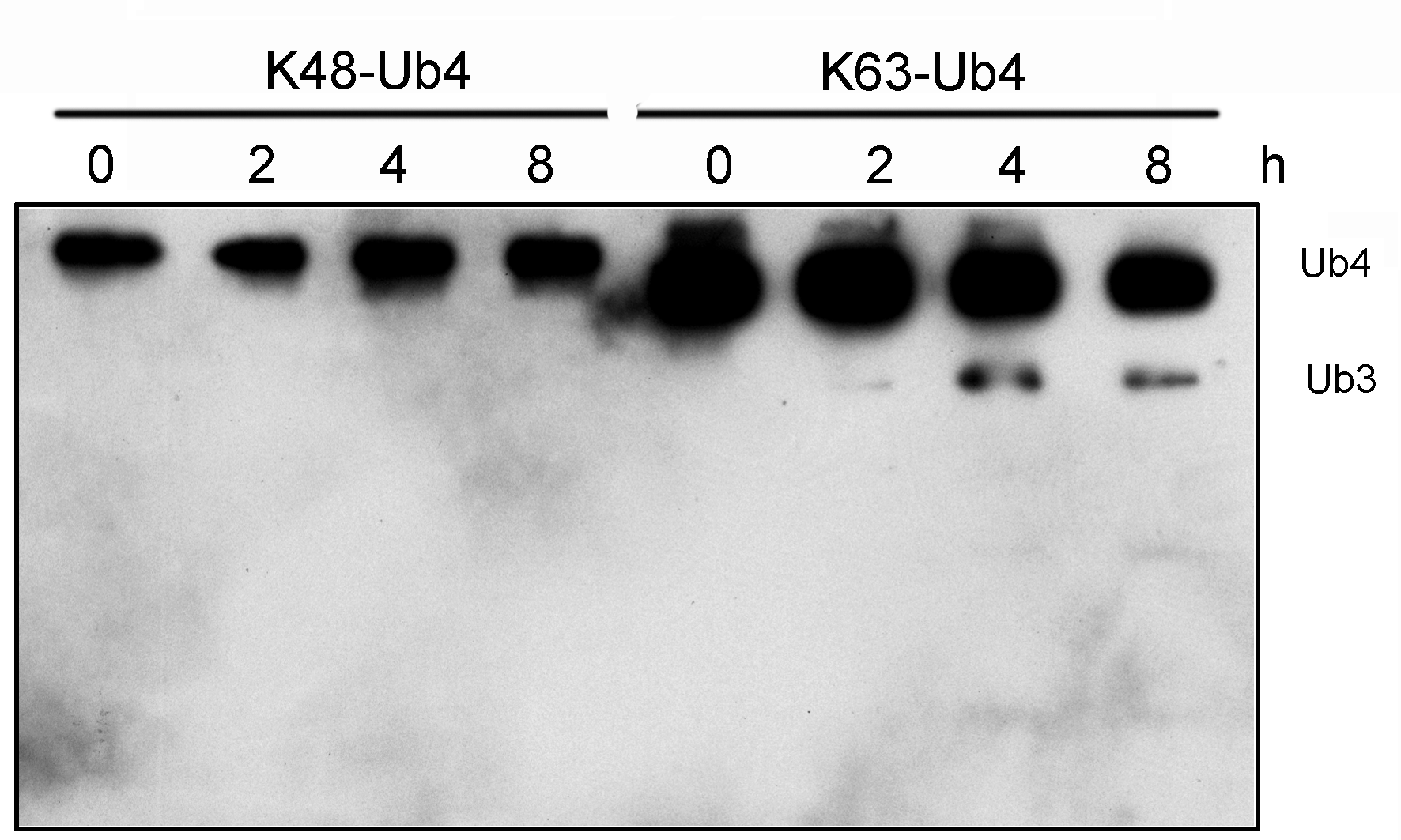


**Figure S4** Time courses of the hydrolysis reactions of USP13 for K48- or K63-linked Ub4 chains. Purified recombinant USP13 (5 μM) were incubated with K48- or K63-linked Ub4 substrate (0.025 μg/μL), and the reaction product in each time point was detected by immunoblotting using an anti-Ub antibody.
