# Supplementary material for: Domain Analysis Reveals That a Deubiquitinating Enzyme USP13 Performs Non-Activating Catalysis for Lys63-Linked Polyubiquitin
Source: PLoS One. 2011 Dec 28;6(12):e29362. doi: 10.1371/journal.pone.0029362 (PMC3247260; doi:10.1371/journal.pone.0029362)
Supplement: Figure S5 — Mass spectrometric analysis of the metal ions in USP13-ZnF. A, Spectrum of USP13-ZnF in native state. The main peak is at a molecular weight of 12889. B, Spectrum of USP13-ZnF in denatured state. The main peak is at a molecular weight of 12823. The peak shift of 66 units (12889–12823) indicates that only one zinc ion (atomic weight of 65) exists in the ZnF domain. (DOC) [file pone.0029362.s005.doc]

**Figure S5**


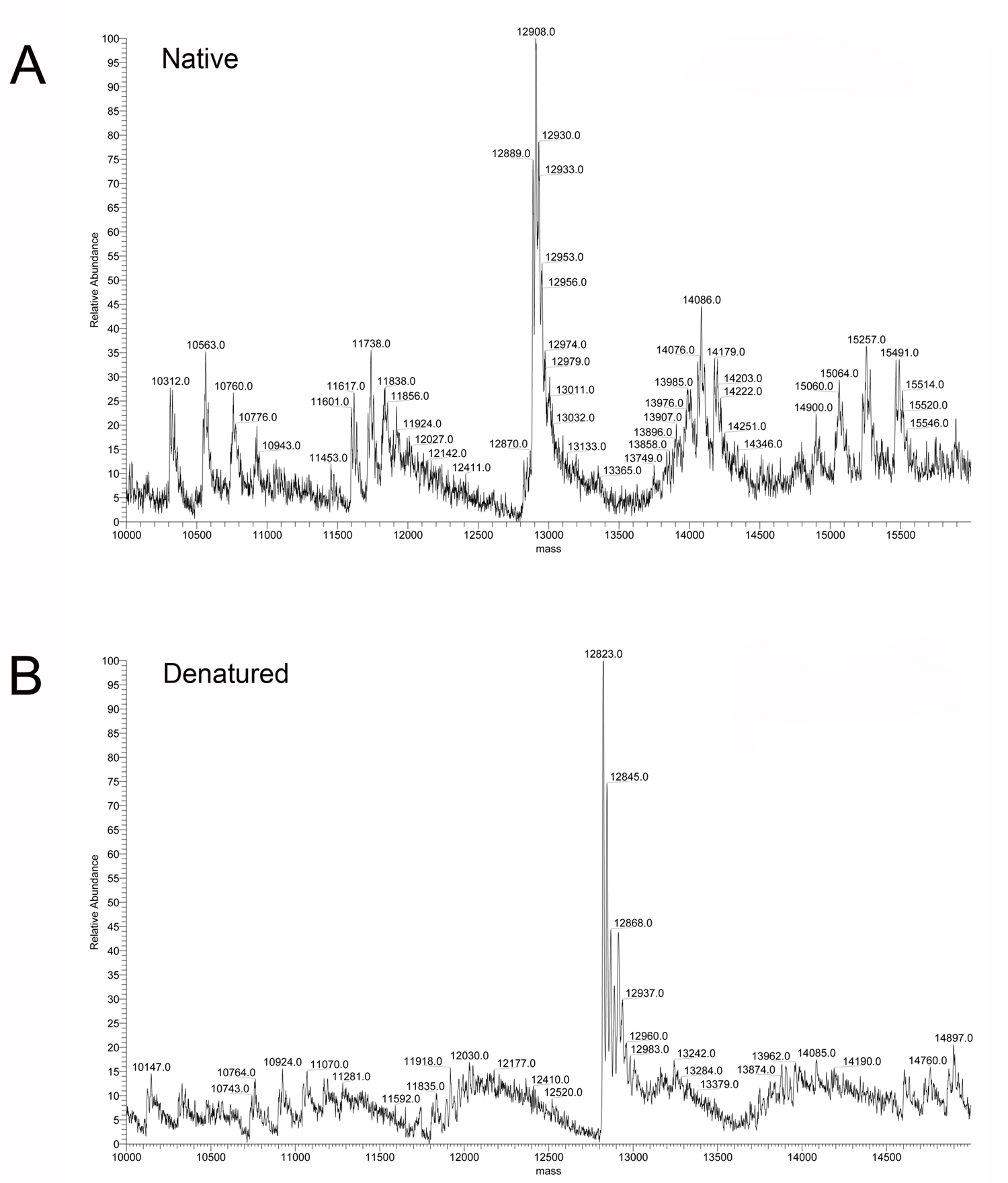


**Figure S5** Mass spectrometric analysis of the metal ions in USP13-ZnF. *A*, Spectrum of USP13-ZnF in native state. The main peak is at a molecular weight of 12889. *B*, Spectrum of USP13-ZnF in denatured state. The main peak is at a molecular weight of 12823. The peak shift of 66 units (12889 – 12823) indicates that only one zinc ion (atomic weight of 65) exists in the ZnF domain.
