# Supplementary material for: Domain Analysis Reveals That a Deubiquitinating Enzyme USP13 Performs Non-Activating Catalysis for Lys63-Linked Polyubiquitin
Source: PLoS One. 2011 Dec 28;6(12):e29362. doi: 10.1371/journal.pone.0029362 (PMC3247260; doi:10.1371/journal.pone.0029362)
Supplement: Figure S6 — ITC experiments for measuring the binding affinities of different USP5-ZnF mutants with Ub. The mutants include R221K, Y261F, R222W, Y223F, R222W/Y223F and R221K/R222W/Y223F. All the titrations were performed under the same condition. The dissociation constants of the mutants binding with Ub were estimated from the titration curves by a 1∶1 stoichiometry. (DOC) [file pone.0029362.s006.doc]

**Figure S6**


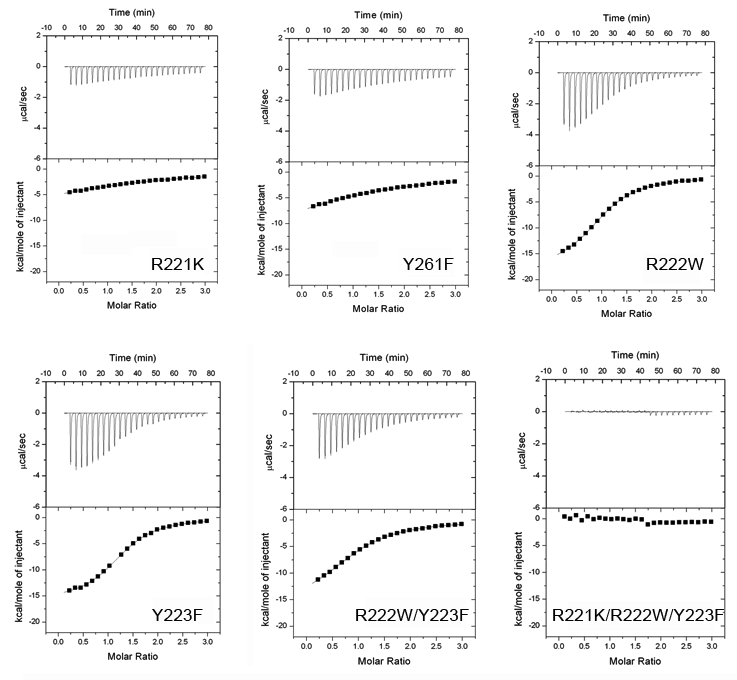


**Figure S6** ITC experiments for measuring the binding affinities of different USP5-ZnF mutants with Ub. The mutants include R221K, Y261F, R222W, Y223F, R222W/Y223F and R221K/R222W/Y223F. All the titrations were performed under the same condition. The dissociation constants of the mutants binding with Ub were estimated from the titration curves by a 1:1 stoichiometry.
