# Supplementary material for: Domain Analysis Reveals That a Deubiquitinating Enzyme USP13 Performs Non-Activating Catalysis for Lys63-Linked Polyubiquitin
Source: PLoS One. 2011 Dec 28;6(12):e29362. doi: 10.1371/journal.pone.0029362 (PMC3247260; doi:10.1371/journal.pone.0029362)
Supplement: Figure S7 — GST pull-down experiments for interactions of the UBA domains from USP13 with Ub and other UbLs. A, GST pull-down analysis of USP13-UBA12 with GST-fused Ub and other UbL proteins. 50% USP13-UBA12 was loaded as a control. The samples were analyzed by SDS-PAGE with Coomassie blue staining. UBA12 from USP13 bind with Ub but not with other UbLs. B, GST pull-down experiment for interactions of the single UBA domains from USP13 with Ub. The GST-fused UBA1 and UBA2 of USP13 were purified from E. coli and incubated with Ub for pull-down analysis. The samples were then analyzed by SDS-PAGE with Coomassie blue staining. 50% Ub was loaded as an input. (DOC) [file pone.0029362.s007.doc]

**Figure S7**


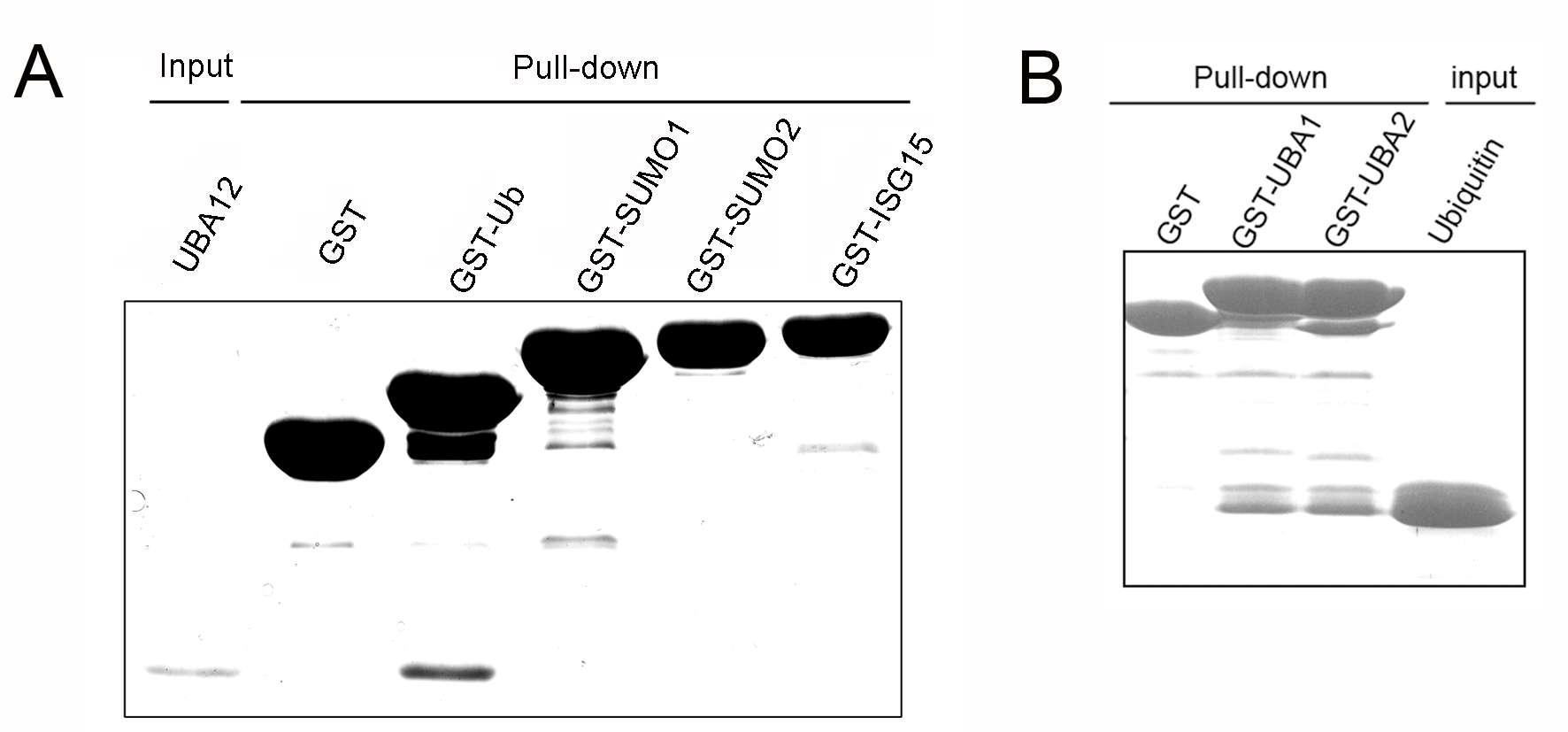


**Figure S7** GST pull-down experiments for interactions of the UBA domains from USP13 with Ub and other UbLs. *A*, GST pull-down analysis of USP13-UBA12 with GST-fused Ub and other UbL proteins. 50% USP13-UBA12 was loaded as a control. The samples were analyzed by SDS-PAGE with Coomassie blue staining. UBA12 from USP13 bind with Ub but not with other UbLs. *B*, GST pull-down experiment for interactions of the single UBA domains from USP13 with Ub. The GST-fused UBA1 and UBA2 of USP13 were purified from *E. coli* and incubated with Ub for pull-down analysis. The samples were then analyzed by SDS-PAGE with Coomassie blue staining. 50% Ub was loaded as an input.
