# Supplementary material for: Domain Analysis Reveals That a Deubiquitinating Enzyme USP13 Performs Non-Activating Catalysis for Lys63-Linked Polyubiquitin
Source: PLoS One. 2011 Dec 28;6(12):e29362. doi: 10.1371/journal.pone.0029362 (PMC3247260; doi:10.1371/journal.pone.0029362)
Supplement: Figure S8 — NMR titration showing that USP13-UBA12 cannot interact with ISG15. Shown is the overlay of 1H-15N HSQC spectra of USP13-UBA12 (0.2 µM) in the absence (red) or presence (cyan, 1: 8) of ISG15. (DOC) [file pone.0029362.s008.doc]

**Figure S8**


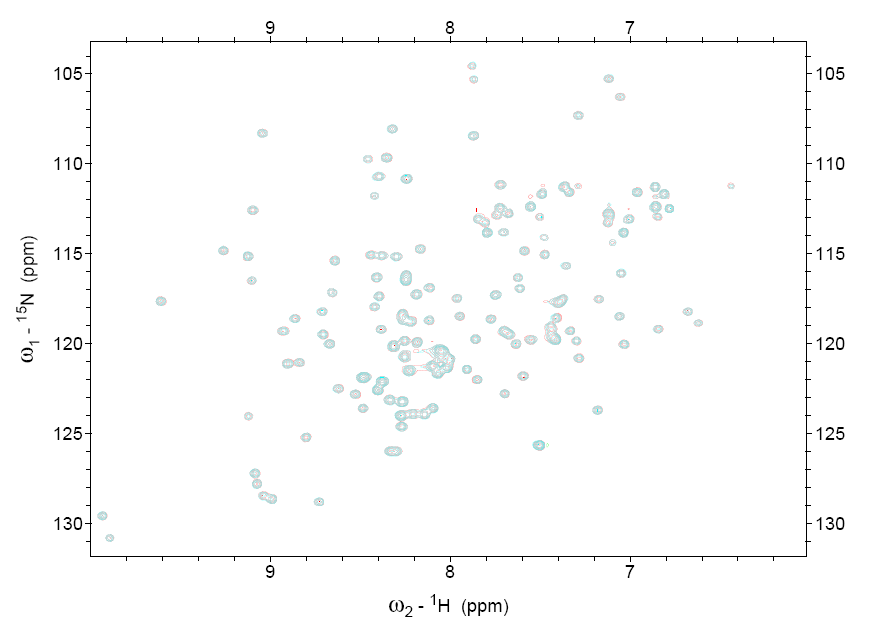


**Figure S8** NMR titration showing that USP13-UBA12 cannot interact with ISG15. Shown is the overlay of ^1^H-^15^N HSQC spectra of USP13-UBA12 (0.2 μM) in the absence (red) or presence (cyan, 1: 8) of ISG15.
