# Supplementary material for: Domain Analysis Reveals That a Deubiquitinating Enzyme USP13 Performs Non-Activating Catalysis for Lys63-Linked Polyubiquitin
Source: PLoS One. 2011 Dec 28;6(12):e29362. doi: 10.1371/journal.pone.0029362 (PMC3247260; doi:10.1371/journal.pone.0029362)
Supplement: Figure S9 — Purification of USP13, USP5 and their mutants. GST-fused USP13 and its C345A mutant were purified by glutathione agarose. USP13, USP5, USP13-5ZnF and the ZnF triple mutant of USP5 (R221K/R222W/Y223F) were purified by Ni-NTA agarose, and the Trx-His6 tags were removed by thrombin cleavage at 22°C overnight, and further purified by Superdex-200 (GE Healthcare). Samples were analyzed by SDS-PAGE with Coomassie blue staining. All the proteins are stored at −70°C in a buffer of 50 mM Tris-HCl, 150 mM NaCl and 5 mM DTT (pH 8.0). (DOC) [file pone.0029362.s009.doc]

**Figure S9**


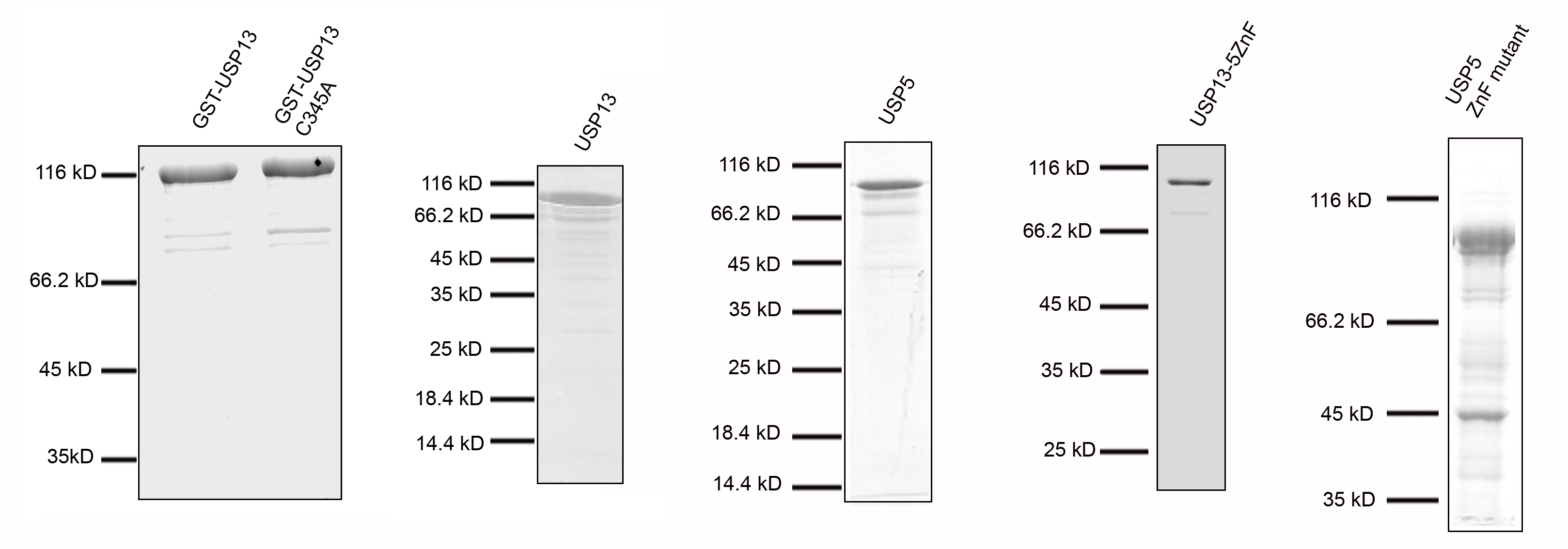


**Figure S9** Purification of USP13, USP5 and their mutants. GST-fused USP13 and its C345A mutant were purified by glutathione agarose. USP13, USP5, USP13-5ZnF and the ZnF triple mutant of USP5 (R221K/R222W/Y223F) were purified by Ni-NTA agarose, and the Trx-His6 tags were removed by thrombin cleavage at 22 ^0^C overnight, and further purified by Superdex-200 (GE Healthcare). Samples were analyzed by SDS-PAGE with Coomassie blue staining. All the proteins are stored at -70 ^0^C in a buffer of 50 mM Tris-HCl, 150 mM NaCl and 5 mM DTT (pH 8.0).
