# Supplementary material for: Domain Analysis Reveals That a Deubiquitinating Enzyme USP13 Performs Non-Activating Catalysis for Lys63-Linked Polyubiquitin
Source: PLoS One. 2011 Dec 28;6(12):e29362. doi: 10.1371/journal.pone.0029362 (PMC3247260; doi:10.1371/journal.pone.0029362)
Supplement: Table S1 — Experimental restraints and structural statistics of the ZnF and UBA12 domains from USP13. (DOC) [file pone.0029362.s010.doc]

**Table S1** Experimental restraints and structural statistics of the ZnF and UBA12 domains from USP13

| Number of experimental restraints | ZnF | UBA12 | |
| --- | --- | --- | --- |
| Total unambiguous distance restraints  Intra residual  Sequential ( | i - j | = 1 )  Medium range (2 **≤** | i – j| **≤** 4)  Long range ( | i – j| ≥ 5 )  Hydrogen bond restraints  Dihedral angle restraints (Φ, Ψ) | 1416  811  287  125  193  42  58, 57 | 1635  786  392  265  192  70  74, 74 | |
| Structure model statistics |  |  | |
| RMSD from experimental restraints  NOE distances (Å)  Dihedral angles (deg.)  RMSD from idealized geometry  Bonds (Å)  Angles (deg.)  Impropers (deg.)  Ramachandran analysis  Residues in most favored regions (%)  Residues in additionally allowed regions (%)  Residues in generously allowed regions (%)  Residues in disallowed regions (%) | 0.030 ± 0.002  0.501 ± 0.108  0.004 ± 0.000  0.549 ± 0.012  1.494 ± 0.071  76.8  21.4  1.0  0.9 | 0.033 ± 0.002  0.515 ± 0.054  0.004 ± 0.000  0.553 ± 0.012  1.489 ± 0.058 | |
|  |  | UBA1 | UBA2 |
|  |  | 86.2  12.9  0.6  0.3 | 80.5  17.1  2.2  0.1 |
| Average atomic RMSDs from the mean structure |  |  |  |
| Secondary structures  Backbone (Å)  Heavy atoms (Å)  All residues  Backbone (Å)  Heavy atoms (Å) | 0.57 ± 0.09  1.11 ± 0.14  1.35 ± 0.20  1.99 ± 0.18 | UBA1 | UBA2 |
|  |  | 0.39 ± 0.08  1.19 ± 0.19  0.68 ± 0.14  1.40 ± 0.16 | 0.11 ± 0.03  0.83 ± 0.21  0.15 ± 0.04  0.90 ± 0.16 |
